# Unraveling Complex Interplay between Heat Shock Factor 1 and 2 Splicing Isoforms

**DOI:** 10.1371/journal.pone.0056085

**Published:** 2013-02-13

**Authors:** Sylvain Lecomte, Léa Reverdy, Catherine Le Quément, Florent Le Masson, Axelle Amon, Pascale Le Goff, Denis Michel, Elisabeth Christians, Yves Le Dréan

**Affiliations:** 1 Transcription, Environment and Cancer group, Institut de Recherche sur la Santé, l’Environnement et le Travail, Inserm U1085, Université de Rennes 1, Rennes, France; 2 Centre de Biologie du Développement, UMR CNRS 5547, Université de Toulouse 3, Toulouse, France; 3 UMR CNRS 6251, Institut de Physique de Rennes, Université de Rennes 1, Rennes, France; Dana-Farber/Harvard Cancer Institute, United States of America

## Abstract

Chaperone synthesis in response to proteotoxic stress is dependent on a family of transcription factors named heat shock factors (HSFs). The two main factors in this family, HSF1 and HSF2, are co-expressed in numerous tissues where they can interact and form heterotrimers in response to proteasome inhibition. HSF1 and HSF2 exhibit two alternative splicing isoforms, called α and β, which contribute to additional complexity in HSF transcriptional regulation, but remain poorly examined in the literature. In this work, we studied the transcriptional activity of HSF1 and HSF2 splicing isoforms transfected into immortalized Mouse Embryonic Fibroblasts (iMEFs) deleted for both *Hsf1* and *Hsf2*, under normal conditions and after proteasome inhibition. We found that HSF1α is significantly more active than the β isoform after exposure to the proteasome inhibitor MG132. Furthermore, we clearly established that, while HSF2 had no transcriptional activity by itself, short β isoform of HSF2 exerts a negative role on HSF1β-dependent *trans*activation. To further assess the impact of HSF2β inhibition on HSF1 activity, we developed a mathematical modelling approach which revealed that the balance between each HSF isoform in the cell regulated the strength of the transcriptional response. Moreover, we found that cellular stress such as proteasome inhibition could regulate the splicing of Hsf2 mRNA. All together, our results suggest that relative amounts of each HSF1 and HSF2 isoforms quantitatively determine the cellular level of the proteotoxic stress response.

## Introduction

Proteasome is a major protein complex responsible for regulated degradation of intracellular proteins, and its activity is modified in many disorders. For example, a decrease in proteasome activity is associated with neurodegenerative diseases, whereas an increase catalytic activity is associated with cancers [1]. Thus, proteasome is a prime target in cancer therapy and bortezomib was the first proteasome inhibitor authorized as anti-tumor agent in humans. In a previous work, we have shown that proteasome subunit expression was regulated by heat shock factors [2]. Heat shock factor 1 (HSF1) and heat shock factor 2 (HSF2) belong to the family of transcription factors, which are essential for the expression of heat shock proteins (Hsps) in response to protein insults. HSF1 is the main factor responsible for Hsp induction, which is abolished in HSF1 deficient cells or organism, and cannot be rescued by HSF2 alone [3]. Among proteotoxic stress, proteasome inhibition, but not heat shock, activates HSF2 [4]. Interestingly, it was shown that after treatment with MG132, a classical proteasome inhibitor, HSF1 and HSF2 can form heterotrimers and bind to DNA [5]. The exact role of such heterotrimers is not yet fully understood, but it was proposed that HSF2 could act as a modulator of HSF1 activity [6]–[7].

Moreover, both HSF1 and HSF2 exist under two different isoforms produced by alternative splicing. This increases the diversity of potential HSF homo and heterotrimers and adds more complexity to HSF regulation. Regarding HSF1, only two publications refer to the existence of two splicing isoforms (Hsf1α and Hsf1β) in mice [8]–[9]. Hsf1α results from a splicing process maintaining the insertion of the exon 11 (66 bp), and thus producing a longer protein in comparison to the β isoform. This exon is flanked by two introns presenting a consensus-splicing site. The 22 amino acids encoded by exon 11 are located in the C-terminal domain, adjacent to the hydrophobic region C (HR-C) that is important to maintain HSF1 under an inactive form in absence of stress signal. This additional region could form a leucine-zipper pattern possibly involved in the temperature activation of HSF1α [9]. Furthermore, it was shown that the relative quantity of both isoforms was regulated in a tissue specific manner in mice, but surprisingly, their transcriptional activities have never been studied so far. Like HSF1, the long and short HSF2 isoforms are named HSF2α and HSF2β, respectively [10]. HSF2α contains also an alternative exon 11, which is similarly flanked by two introns containing consensus-splicing sites. Hsf2 exon 11 encodes an 18 amino acids region located after HR-C and partially overlapping the activation domain called AD-1. The relative quantity of HSF2 isoforms is tissue specific [8], [10]. Activities of HSF2 isoforms have been better documented in studies mainly conducted in K562 erythroleukemia cells, since HSF2 activity is particularly efficient in these cells. Overexpression of HSF2β resulted in a weaker Hsp70 induction compared to HSF2α [11]. This confirmed previous work showing that HSF2α is a more potent transcriptional activator than the HSF2β isoform [10]. However, as K562 erythroleukemia cells contain a mixture of both HSFs in their two existing isoforms, it is difficult to distinguish the actual role of each isoform in the context of heterotrimers.

Since the consequences of proteasome inhibition on HSF1 and HSF2 isoform expression and activity have not yet been fully characterized, we took advantage of existing cells which are deficient for both HSF1 and HSF2. Using defined transfections, we sought to determine the distinct role of each HSF1 and HSF2 isoforms independently of endogenous HSFs. Here, we show that HSF1α and HSF1β exert different transcriptional activity and we provide evidence for a specific repressor role of HSF2β. Finally, our data and analyses establish that the relative quantity of each HSF2 isoforms is regulated in a stress-dependent manner.

## Materials and Methods

### Animals, Embryos and Cell Culture

Mixed genetic background wild type, *hsf1−/−* and *Hsf2^−/−^* mice, previously provided by Dr IJ Benjamin (University of Utah, Salt Lake City) and described elsewhere [12], were used in those experiments. For embryos collection, females were superovulated and mated with males of corresponding genotypes as described in [13]. Plug was considered as day 0.5 after fertilization (0.5 days post coitum (dpc)) and embryos were collected at the 2-cell stage (1.5 dpc) to be cultured to the blastocyst stage as previously described [13]. Blastocysts were obtained on 3–3.5 dpc and used to perform real time RT-PCR.

Immortalized Mouse Embryonic Fibroblasts wild type (WT) or deleted for both *Hsf1* and *Hsf2* (*Hsf1.2^−/−^*) were obtained by intercrossing single knockout mice [12], [14]. The primary cells were then immortalized using SV40 large T antigen by Dr Valérie Mezger (UMR CNRS 7216, Paris, France). Cells were cultivated as previously described [2].

### Plasmid Constructs

pHSE_2x_-TATA-Firefly luciferase was obtained by cloning two synthetic HSEs (5′-GAAgcTTCtaGAAgcTTCtaGAAgcTTC-3′) followed by a TATA box into the pGL3 vector (Promega) at *KpnI* and *SacI* sites. The plasmid pTK-Renilla luciferase (gift from Dr Island, INSERM U991, Rennes, France) was used as control to normalize transfection efficiency. All Hsf isoforms were cloned in pCR3.1 expression vector (Invitrogen). For Hsf1α, the coding sequence was amplified by RT-PCR from total RNA of iMEFs WT, using primers flanking the open reading frame and providing restriction site for *EcoRI* and *NotI*. Then, PCR fragment was cloned to pCR3.1 and the cloned isoform was verified by sequencing. The Hsf1β and Hsf2β cDNAs (in pGEM1-HSF1β and pGEM-HSF2β vector, respectively) were a gift from Dr Richard Morimoto (Northwerstern University, Evanston IL, USA) and they were subcloned in pCR3.1 at *EcoRI* site. The pβactin-HSF2α vector was a gift of Dr Kevin Sarge (University of Kentucky, Lexington KY, USA) and the cDNA was also subcloned in pCR3.1 at *EcoRI* and *NotI* sites.

### Transfection and Luciferase Assay

All transfections were performed using JetPEI (Polyplus Transfection) according to the manufacturer’s instructions. Cells were harvested 24 h after transfection and 8 h of treatment with MG132 at 2.5 µM, or with an equivalent volume of dimethyl sulfoxide (DMSO) as control. For heat shock experiment, cells were maintained at 45°C during 20 min and then transferred at 37°C during 6 h for recovery. Firefly luciferase and Renilla luciferase activities were determined with Dual luciferase reporter assay (Promega).

### RT-PCR Analysis

One million of WT iMEFs were plated on a 10 cm dish and treated with MG132 at 1 µM during 2 h, 4 h, 6 h, 8 h or 10 h. After 10 h of MG132 treatment, cells were allowed to recover for 1 h, 6 h or 10 h, and then harvested. Total RNAs were extracted with TRIzol reagent (Invitrogen) and 5 µg of RNAs were retro-transcribed using M-MLV RT (Invitrogen). To discriminate Hsf2α and Hsf2β, primers flanking alternative exon (*forward:* CATGTCTAGTGCTGTCCAGC and *reverse:* 5′-GAGCTCATCGACTTCTATGG-3′) were used in RT-PCR experiment. RT-PCR of the housekeeping gene Glyceraldehyde-3-phosphate deshydrogenase (Gapdh) (*forward:* 5′-TGAAGCAGGCATCTGAGGG-3′ and *reverse:* 5′-CGAAGGTGGAAGAGTGGGAG-3′) was used as control for the quality of extraction and retro-transcription. Densitometric analysis was made with Quantity One software (Bio-Rad).

RT-real time PCR experiments were performed on blastocysts as described in [15]. Experiments to determine the relative abundance of Hsf2 isoforms used primers to detect the total population of Hsf2 transcripts (Hsf2 *forward:* 5′-AGGGGAGTACAACTGCATCG-3′and *reverse:* 5′- CAGGCGGACAAGCTTACTC-3′) [16] and primers designed to amplify only Hsf2α isoform (*forward:* 5′-AGTTCTGTGCAGATGAATCCCACAG-3′ and *reverse:* 5′-GCAGATGCAGAGTTCCCATCC-3′). Experiments performed to measure Hsp70.1 transcripts used the following primers: Hsp70.1 *forward*: 5′-TTGTCCATGTTAAGGTTTTGTGGTATA-3′, Hsp70.1 *reverse*: 5′-GTTTTTTTCATTAGTTTGTAGTGATGCAA-3′. The experiments were performed at least in duplicate with one or two independent groups of embryos included in each experiment (n = 20 blastocysts). Results were normalized using 18S RNA amplification.

### Protein Extracts and Immunoblot Analysis

To evaluate protein expression after transfection, 200 000 *Hsf1.2^−/−^* iMEFs per well were seeded in a 6-well plate. Cells were transfected as described above and were harvested after 24 h of transient expression and 8 h of treatment with MG132 at 2.5 µM. To analyze the expression of endogenous HSF2, 1 million of WT cells were plated in a 10 cm dish and were treated the next day with MG132 at 1 µM, or with DMSO for 10 h. Whole cells extracts were prepared with NP-40 lysis buffer (50 mM Tris-HCl pH 8, 150 mM NaCl, 10 mM EDTA, 1% NP-40, 0.2% sarkosyl). Proteins were separated on an 8.5% or a 12% polyacrylamide gel to discriminate HSF2 isoforms and transferred to a nitrocellulose membrane (Amersham Bioscience). Anti-HSF1 (4B4) antibody (ab44819), anti-HSF2 (3E2) antibody (sc-13517) and Anti-GFP antibody (a11122) were purchased from Abcam, Santa Cruz Biotechnology and Molecular Probes, respectively.

Four hours of migration at 40 mA were necessary to separate HSF2α and HSF2β. Ponceau staining was performed to assess equal loading in place of classical actin control, which was excluded from the gel during the long electrophoresis.

### Co-immunoprecipitation

*Hsf1.2^−/−^* iMEFs were transfected with pHSF1β-EGFP and/or with pCR3.1-HSF2α, or with pCR3.1-HSF2β. As control, cells were co-transfected with pEGFP and pCR3.1-HSF2α, or pCR3.1-HSF2β. Cells were treated with MG132 at 1 µM during 10 h. Anti-GFP antibody (A11122, Molecular Probes) was used for immunoprecipitation, carried out with Nuclear Complex Co-immunoprecipitation kit (Active Motif), according to manufacturer’s instructions.

## Results

### Transcriptional Activities of HSF1 and HSF2 Splicing Isoforms

**Figure 1:**
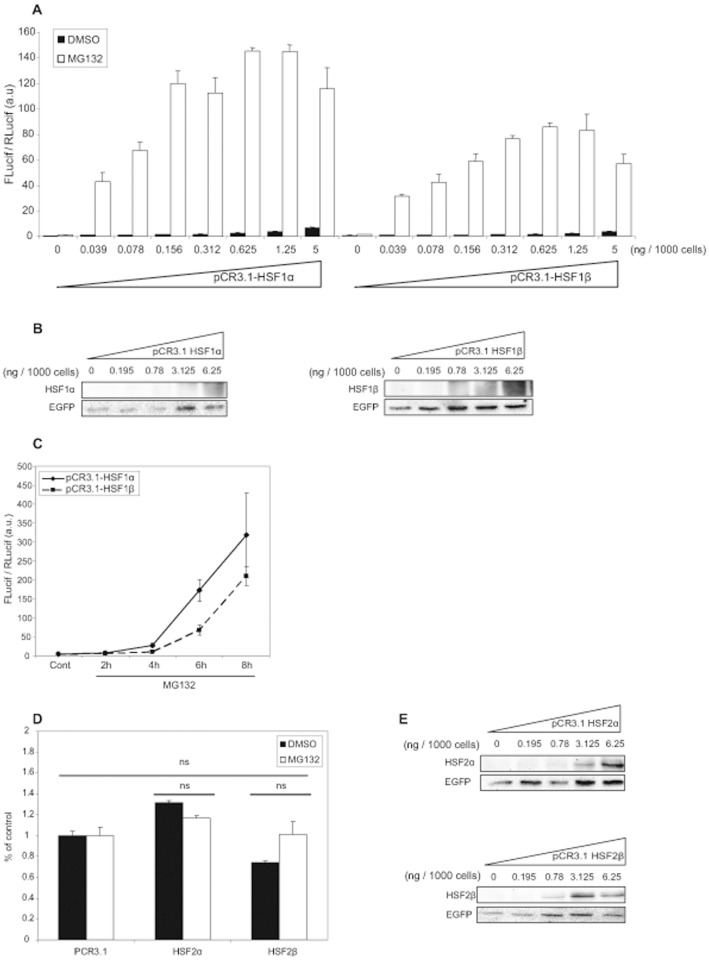
Transcriptional activity of HSF1 and HSF2 isoforms. (A) *Hsf1.2^−/−^* iMEFs were co-transfected with increasing quantity of pCR3.1-HSF1α (left), or pCR3.1-HSF1β (right), in addition to pHSE_x2_-TATA-Luc used as a reporter gene. DNA quantities were adjusted with empty pCR3.1. Transfection efficiency was assessed using the pTK-Rluc reporter gene. Cells were treated with MG132 at 2.5 µM (white) or with DMSO (black) as control, for 8 h. Results correspond to the ratio between firefly luciferase (FLucif) and renilla luciferase (RLucif) activities. The data are from a representative experiment including three independent replicates (mean +/− SD). (B) Representative Western-blot showing the expression of HSF1 isoforms after transfection. *Hsf1.2^−/−^* iMEFs were co-transfected with increasing quantity of pCR3.1-HSF1α (left) or pCR3.1-HSF1β (right) and pEGFP as control for transfection efficiency. Cells were treated with MG132 at 2.5 µM and immunoblots for HSF1 and GFP were performed. (C) *Hsf1.2^−/−^* iMEFs were co-transfected with 12.5 ng of pCR3.1-HSF1α (full line), or pCR3.1-HSF1β (dotted line), with two reporter genes described previously. Cells were treated with MG132 at 2.5 µM or with DMSO, for 2 h, 4 h, 6 h or 8 h. Results correspond to the ratio between firefly luciferase (FLucif) and renilla luciferase (RLucif) activities and are the mean of three independent experiments +/− SD. (D) *Hsf1.2^−/−^* iMEFs were co-transfected with 12.5 ng of pCR3.1-HSF2α, or pCR3.1-HSF2β, with the reporter genes as described in (A). Cells were treated with MG132 at 2.5 µM (black), or with DMSO (white), for 8 h. Results are expressed in percentage of empty vector and represent the mean of three independent experiments +/− SD (Student’s t test, ns: no significant). (E) Representative Western-blot showing the expression of HSF2 isoforms after transfection. *Hsf1.2^−/−^* iMEFs were co-transfected with increasing quantity of pCR3.1-HSF2α (high panel) or pCR3.1-HSF2β (low panel) and pEGFP as control for efficiency. Cells were treated with MG132 at 2.5 µM and immunoblots for HSF2 and GFP were performed.

To characterize the *trans*activation abilities of HSF1 isoforms, we first co-transfected increasing doses of expression vector coding either HSF1α or HSF1β, in *Hsf1.2^−/−^* iMEFs, with pHSE_2x_-TATA-Firefly luciferase as a reporter gene. Then, cells were treated with MG132 at 2.5 µM for 8 h. As presented in Figure 1A, low quantities of HSF1α expression vector provided high level of *trans*activation and HSF1α exhibited 1.4 times higher plateau values than HSF1β. These data suggested that the α isoform was more efficient than the β one to activate HSE-dependent transcription under proteasome inhibition. Nevertheless, increasing quantities of transfected HSF1α vector was accompanied by a more pronounced basal activity (around 10 fold for 5 ng of transfected vector/1000 cells), indicating that the α isoform presents a weak stress-independent activity. To verify that the difference observed in *trans*activation abilities was not due to a defect in the level of expression of the transcription factors, immunoblots were performed with extracts from *Hsf1.2^−/−^* iMEFs that had been co-transfected with an increased quantity of pCR3.1-HSF1α or pCR3.1-HSF1β, in addition to pEGFP used as a transfection efficiency control (Figure 1B). All cells were treated with MG132 at 2.5 µM for 8 h. HSF1α protein (left panel) became detectable when 3.125 ng of vector were transfected per 1000 cells, whereas HSF1β was detected after transfection of a lower amount of vector (0.78 ng/1000 cells), suggesting that the β isoform was more stable than the α isoform, but less effective from a transcriptional point of view. Moreover, both HSF1 isoforms required approximately 80 and 20 fold higher quantity of transfected vector to be detected by immunoblot in comparison to the amount needed to obtain a visible effect by luciferase assay. These results suggest that a very low HSF1 intracellular concentration is sufficient to activate transcription.

To study the kinetic of *trans*activation of each isoform, we co-transfected 12.5 ng of expression vector in *Hsf1.2^−/−^* iMEFs. Then, cells were treated with MG132 at 2.5 µM for 2 h, 4 h, 6 h or 8 h. Stress-dependent induction of transcription was detectable after 4 h of treatment (Figure 1C), showing that both isoforms exhibited similar responses in terms of activation time course.

Finally, HSF2α or HSF2β expression vector were co-transfected with pHSE_x2_-TATA-Firefly luciferase in *Hsf1.2^−/−^* iMEFs. Neither HSF2α nor HSF2β induced the transcription of the HSE reporter gene after treatment with MG132 at 2.5 µM (Figure 1D). Two other reporter genes, containing either HSP70 or p35 gene promoters, were used in similar experiments and gave comparable results since both HSF2 isoforms were unable to significantly trigger Hsp70 or p35 transactivation (data not shown). These data suggest that by itself, HSF2 is a very poor transcription factor, whatever the splicing isoform considered. As previously tested for HSF1, *Hsf1.2^−/−^* iMEFs were co-transfected with increasing quantity of pCR3.1-HSF2α or pCR3.1-HSF2β, in addition to pEGFP used as a transfection efficiency control, to assess the level of expression of the different vectors (Figure 1E). Like for HSF1 isoforms, a minimum of 3.125 ng transfected vector per 1000 cells was necessary to detect HSF2α whereas only 0.78 ng per 1000 cells was required for HSF2β, suggesting that this latter isoform is more stable than HSF2α.

### HSF2β Forms Heterotrimers with HSF1β and Inhibits its Transcriptional Activity

**Figure 2:**
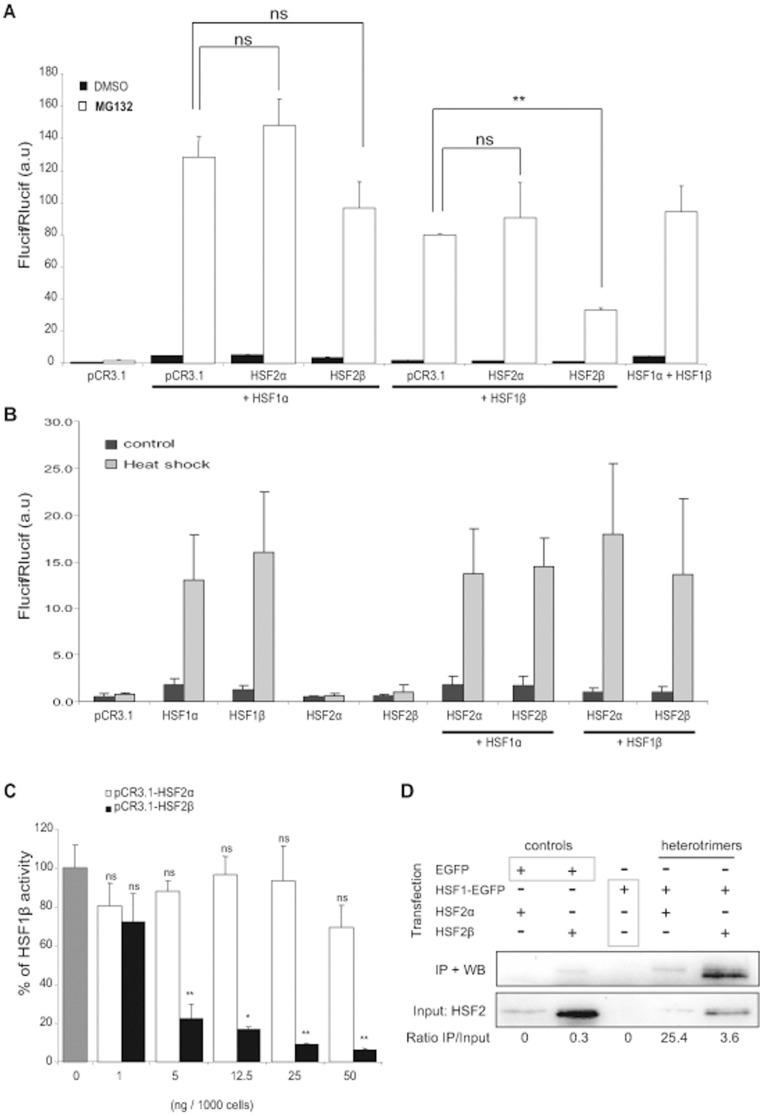
HSF2β interacts with HSF1β and inhibits its transcriptional activity. (A) *Hsf1.2^−/−^* iMEFs were co-transfected with 12.5 ng of pCR3.1-HSF1α or pCR3.1-HSF1β in combination with 12.5 ng of pCR3.1-HSF2α, or pCR3.1-HSF2β. Transcriptional activity was followed with pHSE_x2_-TATA-Luc, and pTK-Rluc was used as control for transfection efficiency. Cells were treated with MG132 at 2.5 µM (white), or with DMSO as control (black). Results correspond to the ratio between firefly luciferase (FLucif) and renilla luciferase (RLucif) activities and are a representative experiment made with three independent replicates (Student’s t test: HSF1α or β alone compared to others conditions. **p<0.01, ns: no significant). (B) Cells were transfected with the indicated expression vectors and the reporter genes, as described in (A). Cells were submitted to heat shock at 45°C for 20 min and put to recovery at 37°C for 6 h (grey). Untreated cells served as control (black). Results are the mean +/− SD of 9 to 12 independent transfections. (C) *Hsf1.2^−/−^* iMEFs were co-transfected with pCR3.1-HSF1β (gray) and with an increase quantity of pCR3.1-HSF2α (white), or pCR3.1-HSF2β (black). Then, cells were treated with MG132 at 2.5 µM for 8 h. Results are expressed in percentage of HSF1β activity and are the mean of three independent experiments +/− SD (Student’s t test: HSF1β alone compare to others conditions. *p<0.05, **p<0.01, ns: no significant). (D) Representative co-immunoprecipitation experiments. *Hsf1.2^−/−^* iMEFs were co-transfected with pEGFP or pHSF1β-EGFP, in combination with pCR3.1-HSF2α or pCR3.1-HSF2β. Cells were treated with MG132 at 1 µM for 8 h and nuclear protein extracts were submitted to EGFP immunoprecipitation, followed by immunoblotting for HSF2. The value of the IP/Input ratio is indicated below the panel.

Previous work from our team had shown that proteasome inhibition was associated with the formation of HSF1/HSF2 heterotrimers [5]. To determine how the different splicing isoforms could impact heterotrimer activity, we co-transfected different combinations of expression vectors with reporter genes in *Hsf1.2^−/−^* iMEFs (Figure 2A). HSF1α transcriptional activity was not statistically different in absence or presence of HSF2 isoforms. Likewise, HSF1β transcriptional activity was not altered when co-transfected with HSF2α. However, it was strongly and significantly decreased (3 fold) after co-transfection with HSF2β. The activity of the heterotrimer was also assessed after heat shock (Figure 2B). When HSF1 isoforms were transfected alone, the same transactivation level was observed whatever the isoform used. As observed after MG132 treatment, HSF2α and HSF2β did not show any significant transcriptional activity under thermal stress. Co-transfection of HSF2 with HSF1 did not affect HSF1 activity. Especially, the combination of HSF1β with HSF2β did not decrease the trimer activity after heat shock, in contrast to MG132 treatment.

To verify that the inhibition observed in MG132 experiment was due to HSF2β itself and not to a side effect related to difference in transfection efficiency, we performed a dose effect experiment (Figure 2C). *Hsf1.2^−/−^* iMEFs were co-transfected with a constant quantity of pCR3.1-HSF1β and increasing doses of pCR3.1-HSF2α or pCR3.1-HSF2β. HSF1β *trans*activation remained similar whatever the amount of co-transfected HSF2α. On the contrary, HSF2β transfection provoked a sharp decrease of *trans*activation with low dose (5 ng/1000 cells), confirming the inhibitory role of HSF2β.

The ability of each HSF2 isoform to associate with HSF1β in response to MG132 treatment was determined by co-immunoprecipitation experiment (Figure 2D). *Hsf1.2^−/−^* iMEFs were co-transfected with expression vector coding either HSF1β-EGFP or only EGFP, in association with HSF2 isoforms. Immunoprecipitation was performed with antibodies against EGFP and blots were probed with HSF2 antibodies. Cells transfected with HSF1β-EGFP, but not HSF2, were used as control and showed no signal after immunoprecipitation, demonstrating that the HSF2 antibodies do not cross-react with HSF1β (lane 3). Interestingly, we found that both HSF2α and β isoforms interact with HSF1β (lane 4 and 5) indicating the formation of heterotrimers. Moreover, HSF2 co-immunoprecipitation required the presence of HSF1, as demonstrated by its absence in the EGFP control (lane 1 and 2).

### Influence of the HSF2β/HSF2α Ratio on HSF1 Transcriptional Activity

**Figure 3:**
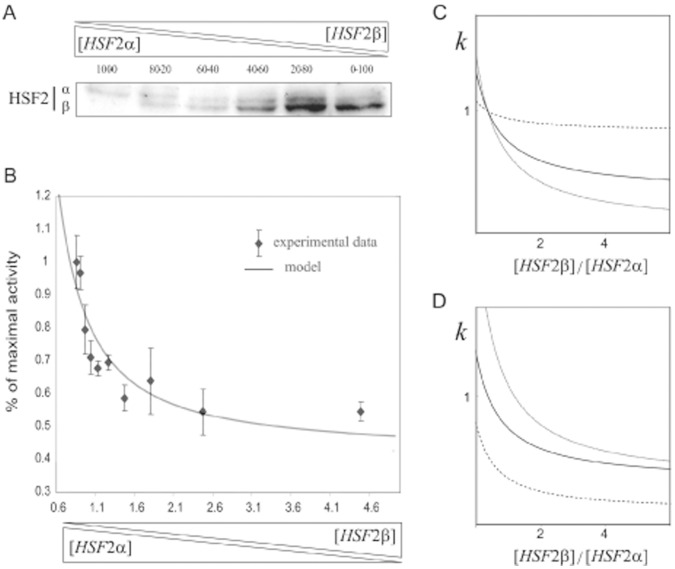
Ratio between HSF2α and HSF2β controls HSF1β transcriptional activity. (A) Representative Western-blot showing the expression of HSF2 isoforms after transfection. *Hsf1.2−/−* iMEFs were co-transfected with pCR3.1-HSF1β and pCR3.1-HSF2α/β to obtain the expression of equivalent amounts of HSF1 and HSF2, and increasing concentration of HSF2β relatively to HSF2α. Cells were treated with 2.5 µM MG132 for 8 h. Protein extracts were loaded on 12% polyacrylamide gel and submitted to a long migration to separate efficiently HSF2 isoforms. HSF2 was revealed by immunobloting. (B) Transcriptional activity induced, with fixed concentrations of HSF1 and total HSF2, but varying combinations of HSF2β/HSF2α. *Hsf1.2−/−* iMEFs were co-transfected with 12.5 ng of pCR3.1-HSF1β and with the same quantity of pCR3.1-HSF2α/β, as previously described. Diamonds correspond to the experimental data obtained in independent triplicates, and expressed as percentage of maximal activity. Solid line drawn to equation 2 with random multimerization and considering that HSF1 is active only in absence of HSF2β in the trimer. (C) HSE-driven transcriptional activities expected from Eq. 2, with a constant and identical amounts of HSF_1_ and total HSF_2_, and when increasing the ratio HSF_2_β/HSF_2_α. The transcriptional strength of a trimer is assumed to be proportional to the number of HSF_1_ monomers present, and the unit of transcriptional strength (*k* = 1) corresponds to that of an HSF_1_ monomer. The strength of trimerization is considered as either (i) identical between hetero- and homodimers (plain line), (ii) 10 fold higher for homodimers (dashed line), or (iii) 10 fold higher for heterodimers (dotted line). (D) HSE-driven transcriptional activities drawn to Eq. 2, with constant and equivalent amounts of HSF1 and HSF2, capable to either randomly homo- or heterotrimerize. Plain line: as for panel C, the transcriptional strength of a trimer is proportional to the number of HSF_1_ monomers included in the trimer and the strength of a HSF_1_ monomer is set to 1 (*k*_3_ = 3 *k*_1_ and *k*_2_ = 2 *k*_1_ and *k*_1_ = 1). Dashed line: Transcriptional strength independent on whether the trimer contains 1, 2 or 3 HSF_1_ monomers (*k*_1_ = *k*_2_ = *k*_3_ = 1). Dotted line: same rule with *k*_1_ = *k*_2_ = *k*_3_ = 3.

To determine how the HSF2β/HSF2α ratio impacts the transcriptional activity of HSF1β, *Hsf1.2^−/−^* iMEFs were transfected with HSF1β and the same amount of total HSF2, including variable HSF2β/HSF2α ratios (Figure 3). HSF2 proteins were first quantified by immunoblotting (Figure 3A) and densitometric analyses. Due to the differences in protein stability of HSF2 isoforms, we calculated from three densitometric analyses a corrective coefficient, linking transfected vector ratio and protein expression. Then, the same transfection conditions were used to measure the transcriptional activity with a luciferase reporter vector (Figure 3B). HSE-driven transcriptional activity diminished when increasing the HSF2β/HSF2α protein ratio. The capacity of HSF2 and HSF1 to heterotrimerize has been evidenced [5], [17] but the stoichiometry of the different HSFs in the trimers remains unknown. Moreover, the kinetic and thermodynamic parameters of HSF trimerization, as well as the precise mode of stepwise HSF assembly, ordered or random, have to be characterized. Hence, in absence of defined data, minimal rules were selected, as listed below. It is considered that HSF2α has no influence on the transcriptional activity of the HSF1 protomers included in a trimer, whereas HSF2β completely inhibits their activity. In addition, HSF trimerization is assumed to occur when bound to DNA, but not in solution. The 3 monomers are supposed to trimerize with microscopic dissociation constants, *K*_1_ or *K'*_1,_ depending on whether they form homo- or hetero-trimer, respectively. For symmetry reasons, one may expect that homotrimers are favored (*K*_1_< *K'*_1_), but considering the close structural relationship between the trimerization domains of the different HSFs, *K*_1_ and *K'*_1_ could in fact be very similar. All types of HSF trimers are supposed to bind to the DNA with the same intrinsic dissociation constant *K*_2_. Global composite constants *K* or *K'* are defined such that *K* = *K*_1_^2^ *K*_2_ and *K'* = *K'*_1_^2^ *K*_2_ (M^3^). There are ten different possible types of trimers, distributing over 27 trimer microstates listed below, so that 11 possible states of HSE can exist, either empty or bound by a HSF trimer. Finally, once bound to DNA, HSF trimers devoid of HSF2β promote transcription from an HSE-driven promoter with transcription rates (time^−1^), depending on the number of HSF1 protomers in the trimer (*k*_1_, *k*_2_ and *k*_3_ for 1, 2 or 3 molecules of HSF_1_ respectively). Let us define the following variables: *x* = [HSF_1_*], the concentration of transcriptionally active HSF1; α = [HSF2α] and β = [HSF2β]_T_. The probability of the different binding states of HSE can be defined as the ratio of their mass action values over the sum of all of them, written Σ.
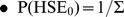


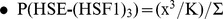


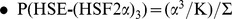


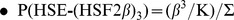


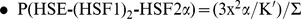


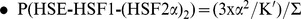


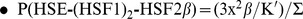


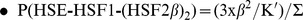


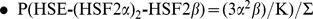


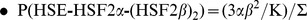


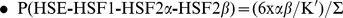



To go further, a ratio of trimerization affinity is written *R* = *K/K*'. In the experiment, the total amount of HSF2 is constant but the ratio of the α and β isoforms is defined by a variable *z* = β/α. The fixed total concentration of HSF2 is *y* = [HSF2]_ T_ = α+β, so that α = *y/*(1+ *z*) and β = *y z/*(1+ *z*). The transcriptional activity (*k*) of the HSE driven promoter can be defined as *k* = Σ *k_i_ P_i_*, where *P_i_* is the probability of presence of the HSF trimer responsible for the maximal transcription initiation rate *k_i_* [18]. Using the rules and values defined above, the global activity can be written after some algebra
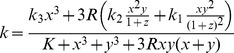
(1)


Finally in the experiment described above, the total concentration of transfected HSF is kept constant (*x*+*y* = *H*). Hence, a variable can be eliminated from Eq. 1, yielding
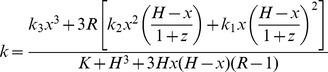
(2)


Eq. 2 satisfactorily matches the experimental profile of the global transcriptional activity obtained when increasing the [HSF2β]/[HSF2α] ratio (Figure 3B). Modifying the relative strength of homo- versus hetero-trimer formation has only marginal influences (Figures 3C, D). Comparison between Figure 3C and Figure 3B suggests that HSF heterotrimers should be stable enough to explain the results, in agreement with the high degree of conservation between the heptad repeat (HR) and the DNA binding domain (DBD) of all HSFs involved in trimerization, regardless of their capacity to initiate transcription.

### Level of Proteasome Activity Regulates the Quantity of Hsf2 Isoforms

**Figure 4:**
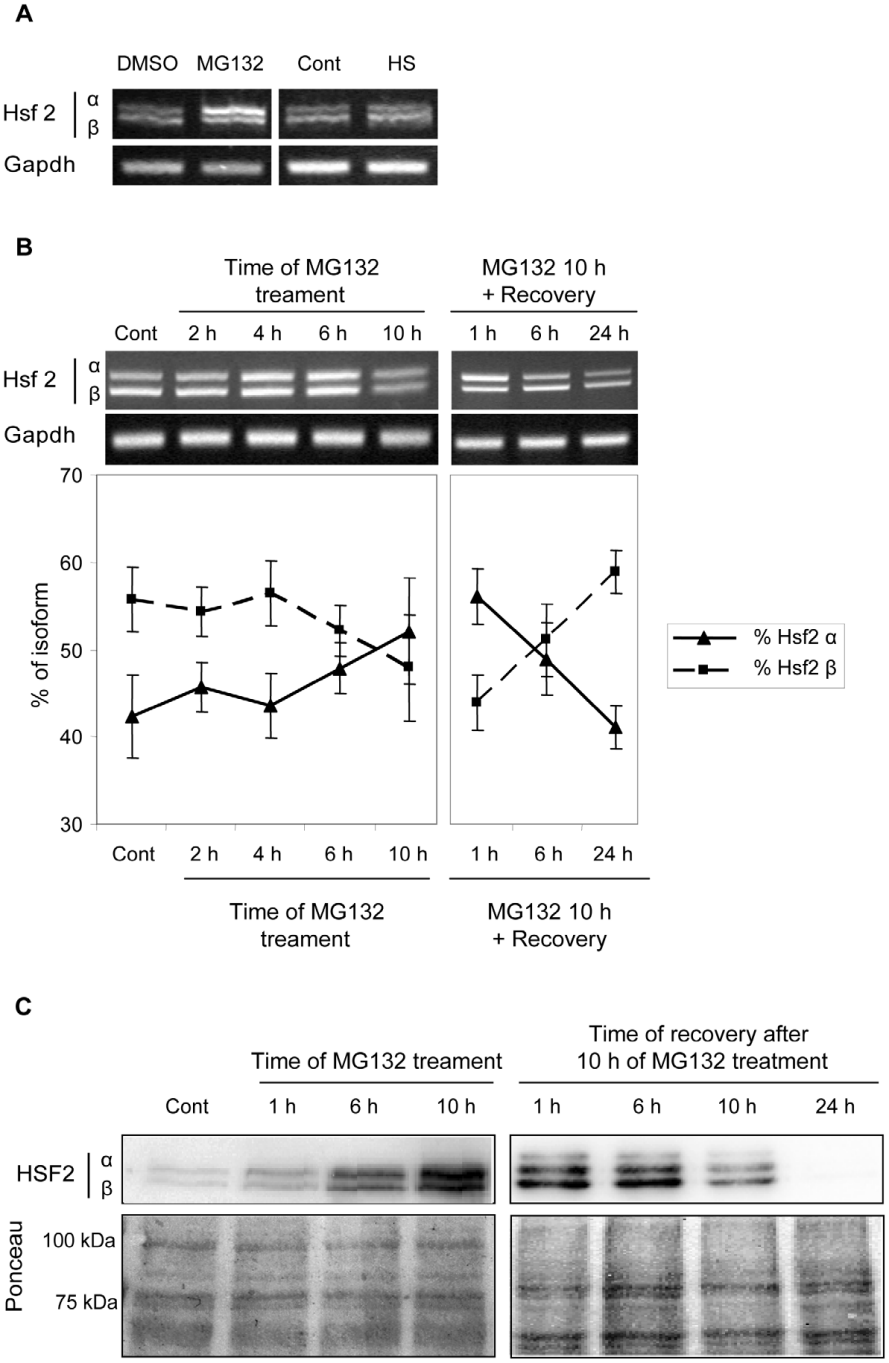
Proteasome inhibition modifies the relative quantity of Hsf2α and Hsf2β at the mRNA level. (A) WT iMEFs were treated with MG132 at 1 µM or with DMSO for 10 h, or were heat shocked 20 min at 45°C and allowed to recover during 6 h. The relative quantity of Hsf2α and Hsf2β transcripts was assessed by RT-PCR using primers flanking the alternative exon. Gapdh RT-PCR served as control for efficient retrotranscritpion and amplification. (B) WT iMEFs were treated during 2 h, 4 h, 6 h or 10 h with MG132 at 1 µM or with DMSO before being harvested. In addition, WT iMEFs exposed to MG132 during 10 h were allowed to recover during 1 h, 6 h or 24 h before collection. Relative quantity of Hsf2α and Hsf2β was assessed by RT-PCR, as described above. Quantity one software (Bio Rad) was used to assess band intensity. Results were expressed in mean of percentage of each isoform +/− SD from four independent experiments. (C) WT iMEFs treated as described in (B) were harvested for protein analysis. Protein extracts were loaded on 12% polyacrylamide gel and submitted to a long migration to separate efficiently HSF2 isoforms. HSF2 was revealed by immunoblotting. Ponceau staining was used to verify the equal loading.

The model described above suggests that the relative concentration of HSF2 isoforms is involved in modulating the HSF1-dependent transcriptional response. So, to assess the relative quantity of each Hsf2 splicing isoforms in cells, RT-PCRs were performed using MG132 treated, or heat shocked WT iMEFs total RNA extracts (Figure 4A). In control and heat shocked cells, Hsf2β was the dominant isoform, whereas in MG132-treated cells, Hsf2α was found to be the major splicing isoform. From these observations, it can be proposed that the way Hsf2 mRNA splicing is regulated depends on the type of stress experienced by the cells. This further implies a Hsf2α to β switch under MG132 treatment. The time course of such switch was analyzed during MG132 exposure (10 h) and after a recovery period (from 1 to 24 h) (Figure 4B). Densitometric analysis showed that Hsf2β represented about 55% of the total Hsf2 transcripts in control cells. After 6 h of treatment, the relative quantity of Hsf2β decreased, whereas the relative quantity of Hsf2α increased to become the dominant isoform (around 55% of the total Hsf2 mRNA). This switch between isoforms is a reversible phenomenon as observed after 24 h of recovery, where the relative quantity of Hsf2α and Hsf2β returned to their initial levels.

The switch in the mRNA isoforms was modest but highly reproducible. Unfortunately, it was more challenging to confirm the isoform switch at the protein level. The WT iMEFs were treated with MG132 at 1 µM for 1 h, 6 h or 10 h and badges of cells were harvested after 1 h, 6 h, 10 h or 24 h of recovery, to detect HSF2 by immunoblot (Figure 4C). In control cells, HSF2α and HSF2β were barely detectable, because HSF2 is a labile protein, constitutively expressed and degraded. During MG132 treatment, HSF2 was highly stabilized which adds an additional level of regulation, in addition to the translation. Differences between isoforms remained difficult to detect because of the lack of sensitivity of the immunoblot method and the absence of isoform specific antibodies.

### Low Hsf2α/Hsf2β Ratio in Blastocyst is Associated with a Weak Response to Proteasome Inhibition

**Figure 5:**
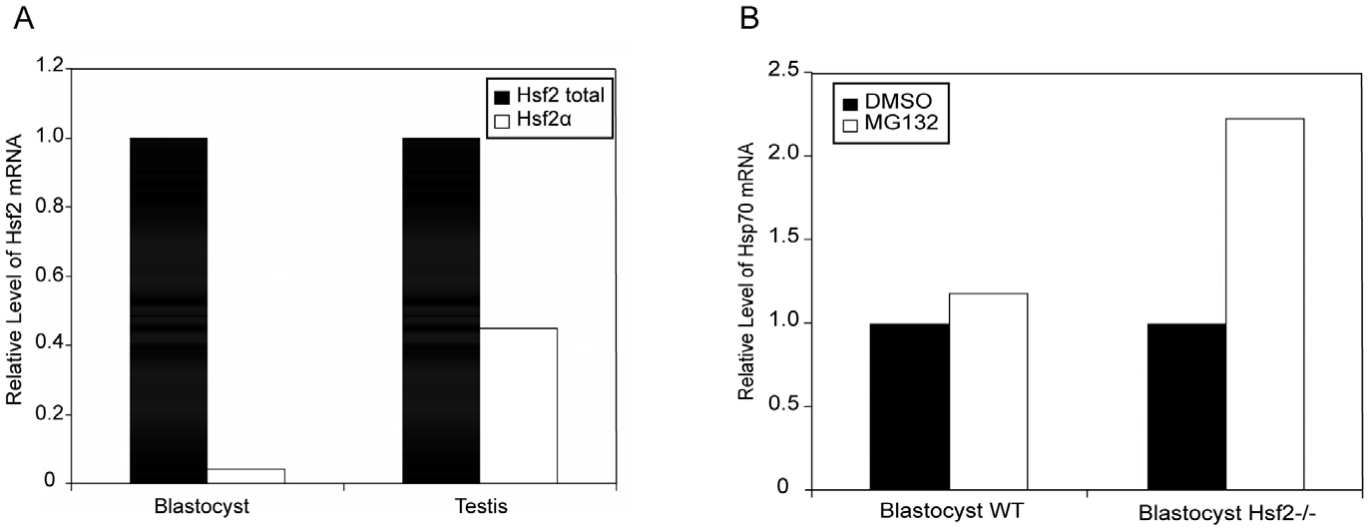
Hsf2 deletion in blastocyst is characterized by a higher Hsp70 induction. (A) Relative quantity of total Hsf2 and Hsf2α transcripts was assessed in blastocyst and testis using RT-real time PCR. Results are expressed as relative expression of Hsf2α compared to total Hsf2 and correspond to the mean of two independent experiments. (B) Hsp70 induction after 4 h of MG132 treatment was assessed by RT-real time PCR in WT and *Hsf2^−/−^* blastocysts. Results are expressed as fold induction of Hsp70 expression after MG132 treatment, normalized to DMSO for each sample, and correspond to the mean of two independent experiments.

HSF2 exhibits a developmentally regulated DNA binding activity at the blastocyst stage (E 3.5 d) [19], which remains unexplored so far. This prompted us to examine the relative abundance of HSF2 isoforms, and the blastocyst response to proteasome inhibition. Due to the limited amount of starting material, we designed primers enabling real time RT-PCR to amplify either the total Hsf2 cDNA, or specifically the α isoform. For comparison, the same strategy was applied to testis total extract (Figure 5A). In blastocysts, Hsf2α represented around 4% of total Hsf2 transcripts (implying that the β isoform would count for 96%), while in testis, the proportion of Hsf2α rose up to 46% of Hsf2 total (Figure 5A). When WT blastocysts were treated with MG132 for 4 h, Hsp70 expression assessed by real time RT-PCR (Figure 5B) was only slightly induced (around 1.2 fold), compared to non-treated ones. On the contrary, in *Hsf2^−/−^* blastocysts, Hsp70 was 2.2 fold induced in MG132 treated versus non-treated embryos. In accordance with our previous results, and speculating that in WT blastocysts where HSF2β was the dominant isoform, the response to proteasome inhibition was blunted. Conversely, in *Hsf2^−/−^* blastocyst where there was no HSF2β protein, the Hsp70 response could be elicited.

## Discussion

Both HSF1 and HSF2 exist as two main splicing isoforms, but very few studies have focused on their respective transcriptional activity. It is uneasy to address this question in cells expressing endogenous HSF1 and 2, because of the coexistence of various HSF complexes, either homo- or hetero-trimers, using potentially both existing isoforms. Therefore, we took advantage of iMEFs genetically depleted for both factors, which provide an invaluable tool to dissect the precise role of each splicing isoforms of HSF1 and HSF2.

We found that HSF1α and HSF1β possessed distinct transcriptional activities, HSF1α being a more potent activator under proteasome inhibition. When HSF1 isoforms were discovered, the authors hypothesized that HSF1α should be activated at higher temperatures than HSF1β, because exon 11 encodes a putative leucine zipper increasing the length of the HR-C domain, known to stabilize HSF1 in the monomeric non-DNA binding form [9]. In our study, we showed that HSF1α had the same kinetic of transcription activation than HSF1β, suggesting that these two isoforms do not differ by their sensitivity to stress intensity but by their transcriptional efficiency. Moreover, this difference in transcriptional competency depends on the type of stress experienced by the cells, since we found that the two isoforms have the same transcriptional activities after heat shock treatment.

Regarding HSF2, previous works in yeast [20] and in K562 erythroleukemia cells [21]–[22] reported a modest, but detectable transcriptional activity for HSF2. It was even suggested that the short HSF2β isoform is a less potent activator than the long HSF2α isoform [10]. Our study revealed that both HSF2 isoforms are transcriptionally inefficient in our cellular model. Taken together, these results suggest that HSF2 transcriptional activity depends upon the cellular context and the presence of indispensable co-factors, or particular stimuli. The absence of HSF2 transcriptional activity also indicates that its main role is not to directly recruit the transcriptional apparatus, but rather to play a pioneer role in chromatin preparation. This hypothesis is in agreement with the fact that HSF2 can prevent compaction of HSF target genes during mitosis [23]. Our study confirmed the role of HSF2 isoforms in the modulation of HSF1 activity [11], [17], [24]. While HSF2α does not affect significantly HSF1 transcriptional activity, HSF2β clearly reduces HSF1 activity. Moreover, this repression is specific to the association of HSF1β with HSF2β, under MG132 treatment. Indeed, it was shown that HSF2 is not activated after heat shock treatment, but rather after inhibition of the ubiquitin-proteasome pathway [4]. Therefore, HSF1-HSF2 heterotrimers can be assembled only after proteasome inhibition, and not after heat shock [5], which might explain why HSF2β inhibitory effect cannot be found after heat shock. According to our results, the presence or absence of a short isoform in the heterotrimer appears to be critical for this modulation of HSF activity. We propose that the leucine zipper conserved in the long isoform could create a novel interaction domain. Thus, this domain could stabilize the activation domain in an optimal conformation, while in the short isoforms, the activation domains could be in a more relaxed conformation, resulting in a less efficient co-factors binding, especially in the context of an HSF1β-HSF2β heterotrimer.

The absence of specific antibody for each isoform, and the technical limitations to accurately determine the ratio of isoforms in a trimer, remain a real concern to further decipher HSF2 mechanism in HSF1 inhibition. In an attempt to alternatively address this question, we have developed a mathematical modelling approach. Our model is different from those previously published on the dynamic of the eukaryotic heat shock response [25]–[27]. In contrast with previous heat shock response models, we did not focus on the various steps involved in the kinetics of Hsp gene activation and its feedback regulation. We rather concentrated on the active HSF trimer that binds to the promoter. We analyzed the impact of the presence of HSF2α and/or HSF2β isoforms on the steady state of HSF1 activity. We postulated that the presence of several HSF isoforms induces the coexistence of different types of heterotrimers. These complexes do not equally transactivate, and they are expected to compete to bind to target promoters. The model we propose satisfies the experimental data obtained by transfection. Based on this model, the strength of the response depends on the proportion of each isoform in the cell. Accordingly, the combination of HSF isoforms synthesized in each cell determines the level of response. This implies that all the cells do not have the same capabilities to respond to proteotoxic stress, and that expression of these HSF isoforms is crucial for the modulation of chaperone expression.

Expression of HSF2 isoforms is tissue-specific. For example HSF2α is the main isoform in testis, while HSF2β is dominant in brain [10]. In addition to this tissue-specific regulation of HSF isoforms, we have shown that the differential splicing of Hsf1 and Hsf2 messenger is also dependent on the type of stress. The switch between the two isoforms is specific to proteasome inhibition, as clearly shown by our RT-PCR experiments.

Taken together our data demonstrate how HSF1 and HSF2 splicing isoforms contribute a new level of complexity to HSF regulation. Our results raise additional questions: how efficient is the proteotoxic response in tissues where HSF1β and HSF2β are dominant? Which evolutionary mechanisms established such complex regulation of the relative quantity of HSF isoforms? It might be critical for stressed cells to possess various ways to modulate HSF1 activity, to achieve better adaptation, and thus survival. Heat shock is an acute stress with massive protein alteration, which requires a rapid and high HSP expression. In that case, HSF1 is found in homotrimeric complex, while HSF2 become insoluble and is found in the perinuclear fraction [28]. On the other hand, proteasome inhibition induces a proteotoxic stress, which can be considered as a chronic stress with the requirement for longer-term adaptation. This should trigger a more limited response, since it could be deleterious for cells or tissues to express excessive amount of HSPs. Proteasome inhibition activates both HSF1 and HSF2 [29], leading to the formation of a diverse population of HSF heterotrimers, with variable transcriptional efficiency [5]. This mechanism could be the best option to control proteotoxic response in cells exposed to in case of chronic stress.
